# SG-APSIC1113: Descriptive study on COVID-19 exposures in Singapore General Hospital

**DOI:** 10.1017/ash.2023.11

**Published:** 2023-03-16

**Authors:** Wee Jin Shawn See

**Affiliations:** Singapore General Hospital, Singapore

## Abstract

**Objectives:** The highly transmissible SARS-CoV-2 has swept across the globe, causing large swaths of COVID-19, displacing medical resources and attention from patients with other life-threatening illnesses, and overwhelming healthcare institutions. Shifting toward endemicity, the Singapore Ministry of Health ceased issuing quarantine orders to close contacts of infected cases on October 11, 2021. However, contact tracing and exposure management within SGH continued with the same risk criteria. We have examined COVID-19 exposures in different hospital locations to determine the effectiveness of surveillance in breaking the chain of transmission. **Methods:** Contact tracing of COVID-19 exposures among Singapore General Hospital (SGH) staff and patients has been conducted since the first COVID-19 diagnosis in January 2020. The information collected is used to identify those at higher risk of infection for enhanced surveillance or isolation. The data analyzed in this study were collected during later periods of the SARS-CoV-2 δ (delta) pandemic wave between August 1, 2021, and December 31, 2021. **Results:** During the 4-month study period, there were 1,686 SARS-CoV-2 exposures in SGH. Among these 1,686 exposures, 1,157 (69%) were contacts with an infected patient. Among these infected source patients, 915 were emergency department patients, 210 were ward inpatients, and 32 were clinic outpatients. The remaining 524 exposure events (31%) were contacts with infected staff, of whom 441 were SGH employees and 83 were employees from other SingHealth institutions. The remaining 5 index cases were visitors to SGH. Of the 1,686 exposure events, 330 had associated at-risk contacts requiring exposure management. Among 330 patient index cases, 213 (64.5%) resulted in 699 exposed contacts (patients vs staff), whereas 117 staff index cases resulted in 435 exposed contacts (patients vs staff). For 434 exposed contacts who were staff, 204 (47%) of their exposures occurred in inpatient ward settings, followed by 153 (35.3%) that occurred in outpatient clinics, 36 (8%) that occurred common lounging areas, 16 (3.6%) that occurred in office sites, 15 (3.4%) that occurred in the community, 8 (1.8%) that occurred in occupation therapy, and 2 (0.5%) that occurred in the emergency department. For 688 exposed contacts who were patients, 579 (84.1%) exposures occurred in inpatient wards, 70 (10.2%) occurred in DEM, 19 (2.7%) occurred in other SingHealth institutions, 16 (2.3%) were exposures to roving porters, and 3 (0.4%) occurred in the community. During the study period, 3 hospital clusters were identified and investigated, one of which included secondary cases. **Conclusions:** Most SARS-CoV-2 exposures in SGH occurred in inpatient settings where patients were index cases. Despite intensive contact tracing and stringent surveillance and isolation measures, inpatient clusters could not be prevented.

